# Supplementary material for: Valley-locked waveguide transport in acoustic heterostructures
Source: Nat Commun. 2020 Jun 12;11:3000. doi: 10.1038/s41467-020-16843-z (PMC7293314; doi:10.1038/s41467-020-16843-z)
Supplement: Supplementary file 1 — Supplementary Information [file 41467_2020_16843_MOESM1_ESM.pdf]

Supplementary Information

Valley-locked waveguide transport in acoustic heterostructures

Wang et al.

Supplementary Note 1. Waveguides A|B$_x$|C, A|B$_x$|A, A|air|C, and C|B$_x$|A

Supplementary Figure 1a shows the dispersion relation of waveguide A|B$_x$|C as a function of x from x=0, i.e., the case of the A|C domain wall, to x=21 (only the half containing the K valley is given for each case, as the other half with the K′ valley is analogous). Supplementary Figure 2 shows the structures and corresponding projected band structures for the heterostructure waveguide A|B$_x$|A (x=11) and the waveguide A|air|C. As shown in Supplementary Figure 2f, the waveguide has several modes in the bandgap of A and C, which can be understood as follows. Denote the width of the waveguide by L and the dispersion relation of sound in air by $\omega = ck$, where $c = 349$ m/s is the sound speed. At frequency f, $k = \frac{\omega}{c} = \frac{2\pi f}{c}$, and the mode number can be estimated as $n = int\left(\frac{k}{\frac{\pi}{L}}\right) + 1 = int\left(\frac{2fL}{c}\right) + 1$, where '*int*' means taking an integer. For the width of the waveguide corresponding to $x = 11$ in A|B$_x$|C, $L = x\frac{\sqrt{3}}{2}a$, $n = 11$ can be evaluated in the bulk gap region of A or C, consistent with that observed in Supplementary Figure 2f. Obviously, n increases with increasing L in the waveguide. However, in the waveguide of the A|B$_x$|C or C|B$_x$|A heterostructure, B has the dispersion relation $\omega - \omega_D = \pm c_D k$ at K and K′, where $\omega_D = 2\pi f_D$, with $f_D = 5.515$ kHz being the frequency at the Dirac point, and k is measured from K or K′. Similarly, at frequency f, $k = \left|\frac{\omega - \omega_D}{c_D}\right| = \frac{2\pi|f - f_D|}{c_D}$, with $c_D = 194.8\, m/s$ (extracted from the band structure of B), and mode number $n = int\left(\frac{k}{\frac{\pi}{L}}\right) + 1 = int\left[\frac{2|f - f_D|L}{c_D}\right] + 1$. Obviously, for x=11 and f falling in the bulk gap of A or C, $n = 2$ can be estimated, that is, only the zeroth- and first-order modes, the 0^+th and 1^+st for the branch $\omega - \omega_D = ck$ and the 0^-th and 1^-st for the branch $\omega - \omega_D = -ck$, exist, as shown in Supplementary Figure 2c and Fig. 1c. For smaller x, for example, $x = 6$, $n = 1$ can be evaluated, which means that in this case, only the zeroth-order modes, i.e., the 0^+th and 0^-th, can be seen in the gap region. However, with increasing thickness L for $x > 11$, higher-order modes, e.g., the 2nd-order modes, enter the gap region of A and C, as shown in Supplementary Figure 1.

The zeroth- and higher-order modes of the two branches in waveguide A|B$_x$|A,

A|B_x|C or C|B_x|A are gapped because of the confinement and modulation by the rough walls of the A and/or C domains, as shown in Supplementary Figure 2c, Fig. 1c and Supplementary Figure 3, indicated by the gray color. However, in waveguides A|B_x|C and C|B_x|A, due to the bulk (A and C) and boundary (B) correspondence, as discussed in the main text, additional waveguide modes appear, as shown in Fig. 1c and Supplementary Figure 3, which are gapless, traversing the gap. The gap of the zeroth-order modes in A|B_x|C (also in A|B_x|A or C|B_x|A) narrows with increasing x, as shown in Fig. 1d. This is understandable because the modulation of the modes by the walls of A and C becomes weaker as the waveguide width increases, and the gap will finally close, restoring the Dirac dispersion of B, when the waveguide is sufficiently wide. In contrast, when x is small, the modulation due to the walls is strong, resulting in a wide gap; when x approaches 0, the gap will finally overlap with the bulk gap, and the zeroth-order modes merge into the bulk bands of A and C.

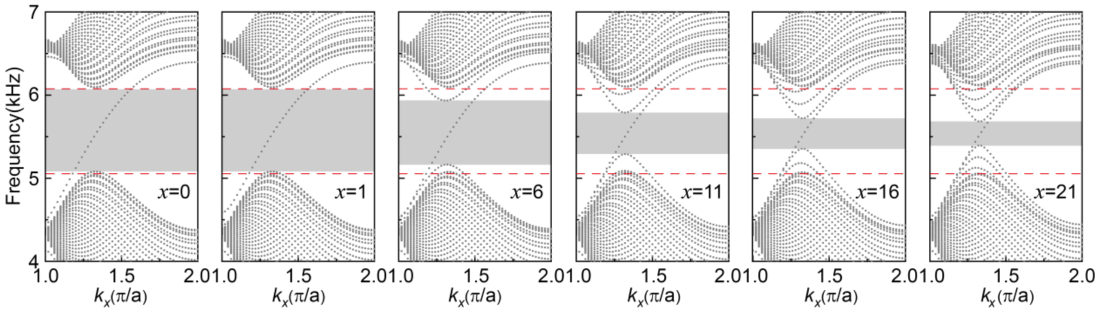

Supplementary Figure 1 | Projected band structures of A|B$_x$|C for x=0, 1, 6, 11, 16, and 21; for each, only the half containing the K valley is shown.

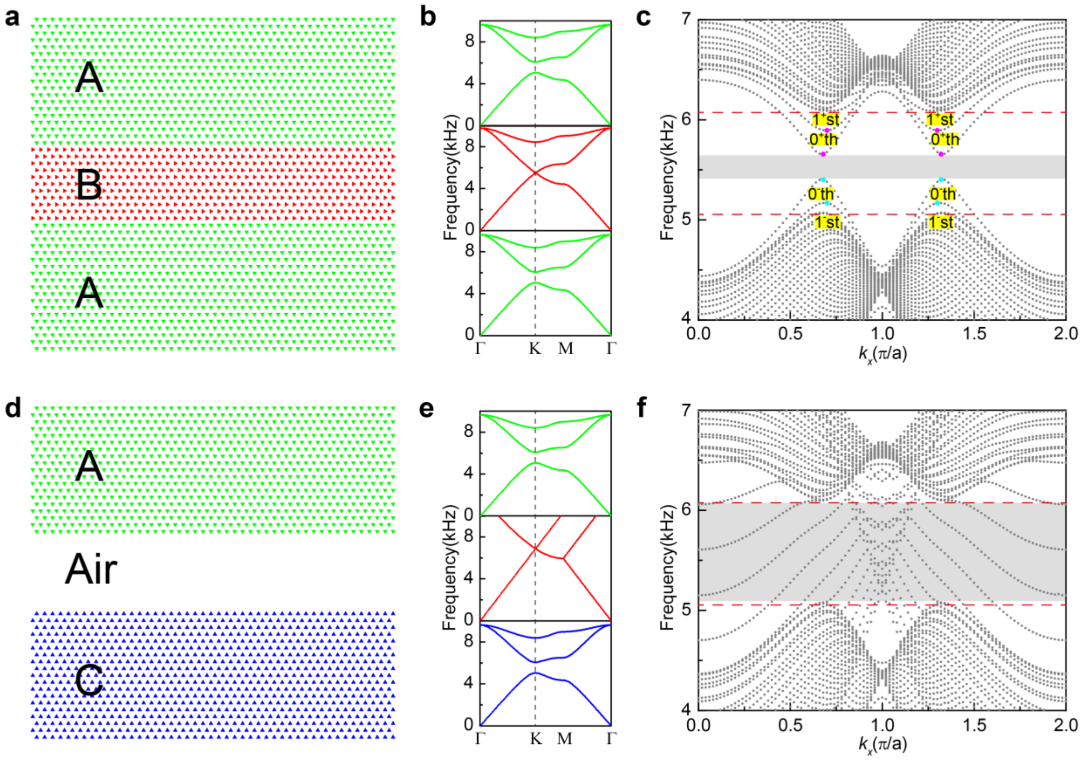

Supplementary Figure 2 | Different heterostructures and corresponding band structures. a-c, A|B_{11}|A structure, the corresponding band structure of each domain, and the projected band structure of the structure. No TVWSs exist in **c**. **d-f**, A|air|C structure derived by removing domain B from A|B_{11}|C shown in Fig. 1a, the corresponding band structure of each domain, and the projected band structure of A|air|C. A number of usual guiding states in air can be observed in **f**.

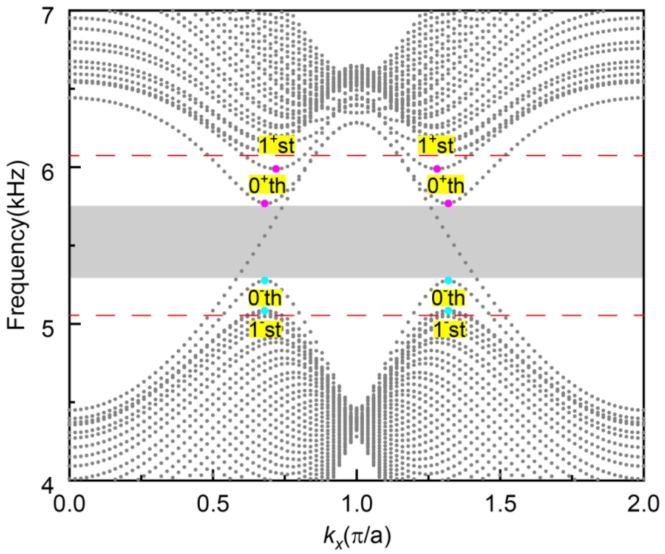

Supplementary Figure 3 | Projected band structure for waveguide C|B_{11}|A, which is nearly the same as that for A|B_{11}|C.
